# Using second harmonic generation to predict patient outcome in solid tumors

**DOI:** 10.1186/s12885-015-1911-8

**Published:** 2015-11-24

**Authors:** K. Burke, M. Smid, R. P. Dawes, M. A. Timmermans, P. Salzman, C. H. M. van Deurzen, David G. Beer, J. A. Foekens, E. Brown

**Affiliations:** 1 Department of Biomedical Engineering, University of Rochester, 207 Robert B. Goergen Hall, Box 270168, Rochester, NY 14627 USA; 2 Department of Medical Oncology, Erasmus MC Cancer Institute, Erasmus University Medical Center, Rotterdam, Netherlands; 3 Neuroscience Graduate Program, University of Rochester, 601 Elmwood Ave, Rochester, NY 14642 USA; 4 Department of Biostatistics and Computational Biology, University of Rochester, 601 Elmwood Ave, Rochester, NY 14642 USA; 5 Department of Pathology, Erasmus Medical Center, Rotterdam, The Netherlands; 6 Departments of Surgery and Radiation Oncology, University of Michigan, Ann Arbor, MI 48109 USA; 7 Department of Neurobiology and Anatomy, University of Rochester, 601 Elmwood Ave, Rochester, NY 14642 USA

**Keywords:** Cancer, Collagen, Second harmonic generation, F/B ratio, Prognosis

## Abstract

**Background:**

Over-treatment of estrogen receptor positive (ER+), lymph node-negative (LNN) breast cancer patients with chemotherapy is a pressing clinical problem that can be addressed by improving techniques to predict tumor metastatic potential. Here we demonstrate that analysis of second harmonic generation (SHG) emission direction in primary tumor biopsies can provide prognostic information about the metastatic outcome of ER+, LNN breast cancer, as well as stage 1 colorectal adenocarcinoma.

**Methods:**

SHG is an optical signal produced by fibrillar collagen. The ratio of the forward-to-backward emitted SHG signals (F/B) is sensitive to changes in structure of individual collagen fibers. F/B from excised primary tumor tissue was measured in a retrospective study of LNN breast cancer patients who had received no adjuvant systemic therapy and related to metastasis-free survival (MFS) and overall survival (OS) rates. In addition, F/B was studied for its association with the length of progression-free survival (PFS) in a subgroup of ER+ patients who received tamoxifen as first-line treatment for recurrent disease, and for its relation with OS in stage I colorectal and stage 1 lung adenocarcinoma patients.

**Results:**

In 125 ER+, but not in 96 ER-negative (ER-), LNN breast cancer patients an increased F/B was significantly associated with a favorable MFS and OS (log rank trend for MFS: *p* = 0.004 and for OS: *p* = 0.03). On the other hand, an increased F/B was associated with shorter PFS in 60 ER+ recurrent breast cancer patients treated with tamoxifen (log rank trend *p* = 0.02). In stage I colorectal adenocarcinoma, an increased F/B was significantly related to poor OS (log rank trend *p* = 0.03), however this relationship was not statistically significant in stage I lung adenocarcinoma.

**Conclusion:**

Within ER+, LNN breast cancer specimens the F/B can stratify patients based upon their potential for tumor aggressiveness. This offers a “matrix-focused” method to predict metastatic outcome that is complementary to genomic “cell-focused” methods. In combination, this and other methods may contribute to improved metastatic prediction, and hence may help to reduce patient over-treatment.

## Background

Breast cancer is the leading cause of cancer related mortality in women [1], predominantly due to metastasis [2]. After surgical resection of the primary tumor, the clinician must choose adjuvant therapy based upon the metastatic potential. Due to their aggressive biological behavior, ER-negative (ER-) tumors are treated with chemotherapy in the majority of patients. However, in ER+ patients whose cancer has not yet spread to the lymph nodes (LNN), the choice between hormonal therapy alone, or in combination with chemotherapy, is more uncertain. Following current standard of care, it is estimated that 40 % of these patients will be “over-treated”, receiving chemotherapy even though they would *not* go on to develop metastatic disease, causing many to endure the emotional distress and severe side effects accompanying chemotherapy [3]. As such, there is a pressing clinical need to accurately predict which ER+, LNN patients have a lower metastatic potential and thus can be spared from over-treatment.

Metastatic potential and treatment response can be predicted to varying degrees of accuracy using traditional histopathology, gene expression measurements [4–8], immunohistochemistry of gene related protein products [9, 10], mass-spectrometry based protein levels [11], image analysis of cell-stromal interactions within the tumor [12], and various other techniques. These techniques provide insights into neoplastic cell function, however, implicit in Steven Paget’s “Seed and Soil” hypothesis is the idea that metastasis involves interactions between tumor cells and their microenvironment [13]. Therefore, we have explored the possibility that the tumor extracellular matrix, specifically the structure of individual collagen fibers as quantified with second harmonic generation microscopy, may provide additional information on tumor metastatic ability.

**Fig. 1:**
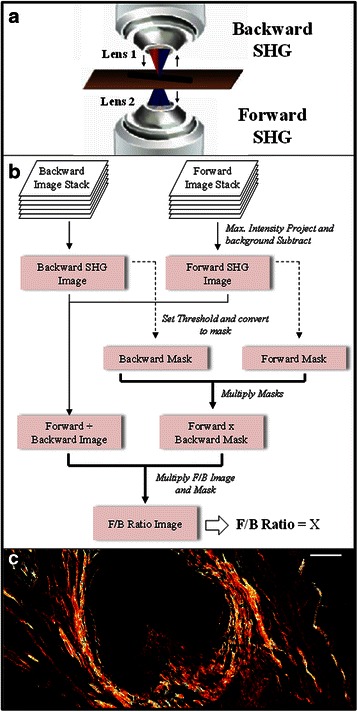
Methodology diagrams. **a** A depiction of the forward- and backward-propagating SHG signal. Red excitation light is focused into the sample by objective lens 1, then SHG is emitted in the backwards direction (towards lens 1) or the forward direction (towards lens 2). **b** A flowchart of the methodology used to analyze SHG images and calculate the F/B ratio. **c** An F/B image of one patient sample. Scale bar is 50 μm

SHG is an intrinsic optical signal in which two incoming photons scatter off of material, producing one emission photon of half the incoming wavelength (Fig. 1). In tumors, SHG is generated by fibrillar collagen and is sensitive to the microscopic structure of the scattering material. Hence SHG emission directionality is sensitive to the diameter of the fibrils that are bundled into collagen fibers, as well as their spacing within the fiber, and the disorder in their packing [14–16]. The ratio of the forward-emitted to backward-emitted SHG (where “forward” is the direction of the incident excitation laser) is known as the F/B ratio and is sensitive to these structural properties of collagen fibers (Fig. 1) [14–16]. Note that these structural properties are intrinsic properties of individual fibers, as opposed to the overall orientation distribution, and its anisotropy, of ensembles of fibers [17]. We have shown that the average F/B of patient biopsy samples can differentiate healthy and breast tumor tissue, and changes with tumor grade and stage [18]. Since SHG is an intrinsic optical signature, measurements of F/B can be performed on typical pathology slides without additional contrast reagents. Furthermore, determination of the average F/B in a sample involves only a straightforward, automated application of pixel intensity analysis that does not require a trained observer. Therefore F/B analysis is an attractive candidate to apply to the prediction of tumor aggressiveness. Here we show that F/B can predict MFS in ER+, LNN breast cancer patients. Similar automated analysis can be performed on the larger scale spatial anisotropy of the orientation of the multiple collagen fibers in these SHG images by performing FFT image analysis [17], therefore for comparison we evaluated the predictive ability of that method as well and found no significant predictive relationship. Based upon its predictive ability in ER+ LNN patients we next investigated F/B in breast cancer patients treated with tamoxifen in a recurrent setting, and found that F/B is also associated with shorter PFS. We further show that the F/B was related to OS in stage I colorectal adenocarcinoma, pointing to the possibility that collagen structure, as reported on by the F/B, and tumor metastatic capacity are linked in both tumor types.

## Methods

### Patient samples

Three-hundred and 44 human breast tumor samples were used from a collection at the Erasmus Medical Center (Rotterdam, Netherlands), which were primarily from one breast cancer genetic expression study [5] and later supplemented by 58 additional ER- samples [19]. These fresh-frozen tissues were initially processed for microarray analysis, and were at a later stage processed for inclusion on a tissue-microarray (TMA) in cases where formalin-fixed paraffin embedded tissues were available as well. Initial sample acquisition was performed in the context of routine measurement of ER and PgR by biochemical assays. The studies on secondary use of archived tissues was approved in writing by the Medical Ethics Committee of the Erasmus Medical Center Rotterdam, The Netherlands (MEC 02.953) and was performed in accordance to the Code of Conduct (The Code for Proper Secondary Use of Human Tissue) of the Federation of Medical Scientific Societies in The Netherlands (http://www.federa.org/codes-conduct). Such secondary use did not require informed consent. All patients were LNN and had not been treated with neoadjuvant nor adjuvant therapy. This allowed for the study of the natural course of the disease and pure tumor aggressiveness, without potentially being confounded by systemic therapy. Some patients received radiation therapy, which has been shown not to affect distant metastases [20], our main focus of this study. The median patient age was 52 years. Follow-up data was recorded every 3 months for 2 years, every 6 months for years 3–5, and every 12 months afterwards. All samples were collected in triplicate as 5 μm thick, 0.5 mm diameter core tissue samples and mounted as TMA slides, in which the uniform tumor presence was verified by hematoxylin and eosin (H&E) staining. Note that the presence of H&E staining does not affect the reported F/B (15), but that the effects of possible variation in time between excision from patient and fixation, as well as the effects of possible variation in time of fixation, are not known and those times are not recorded for the data sets studied here. Patients were tested for ER and progesterone receptor (PgR) status using immunohistochemistry, where the cutoff for receptor positivity was 10 % positive tumor cells. Bloom and Richardson grade and HER2 status data were assessed as described [21] and were available as well for the tissues included in the TMA. In total, 221 TMA-cases were eligible for analysis of F/B ratio, of which 125 were ER+ and 96 were ER-.

Stage I colorectal adenocarcinoma samples were purchased from Yale Tissue Pathology Services (YTMA-8, New Haven Connecticut). Samples were processed as a TMA with one 5 μm thick, 0.5 mm diameter sample per patient, unstained, from within the primary tumor. Samples were collected from 1970–1982 with up to 31 years of follow-up data, resulting in a total of 69 stage I primary colorectal tumors. Lung adenocarcinoma samples were acquired at the University of Michigan, providing a total of 55 stage I lung adenocarcinoma cases [22]. Written subject consent and approval of the Institutional Review Board of the University of Michigan Medical School were obtained to collect specimens from patients undergoing resection for cancer at the University of Michigan Medical Center (Ann Arbor MI) from 1994–2000. All patients underwent the same treatment, surgical resection with intra-thoracic nodal sampling. The lung adenocarcinoma samples were provided as a 5 μm thick section through the full diameter of the tissue. Analysis of H&E stained samples by a trained clinical pathologist was used to ensure images were taken within the tumor proper.

### Imaging

A Spectra Physics MaiTai Ti:Sapphire laser (circularly polarized, 810 nm, 100 fs pulses at 80 MHz) was directed through an Olympus Fluoview FV300 scanner. This was focused through an Olympus UMPLFL20XW water-immersion lens (20×, 0.95 NA), which subsequently captured backward propagating SHG signal. This SHG signal was separated from the excitation beam using a 670 nm dichroic mirror, filtered using a 405 nm filter (HQ405/30 m-2P, Chroma, Rockingham, Vermont), and collected by a photomultiplier tube (Hamamatsu HC125-02). The forward scattered SHG was collected through an Olympus 0.9 NA condenser, reflected by a 565 nm dichroic mirror (565 DCSX, Chroma, Rockingham, Vermont) to remove excitation light, filtered by a 405 nm filter (HQ405/30 m-2P, Chroma, Rockingham, VT) and captured by photomultiplier tube (Hamamatsu HC125-02). During acquisition of the daily calibration sample, a dilute fluorescein isothiocyanate (FITC) solution, a 535/40 filter (535/40 m-2P, Chroma, Rockingham, VT) replaced the 405 nm filters. Forward- and backward-scattered SHG images were simultaneously collected as a stack of 11 images spaced 3 μm apart, with a 660 μm field of view. Imaging conducted on TMA slides of H&E stained, 0.5 mm diameter breast cancer and colon cancer samples permitted one image stack at the center of each sample. For the larger (approximately 3 cm wide) lung cancer samples, 3 locations were chosen randomly in each sample and the 3 resultant F/B values (see below) were averaged.

### F/B image analysis

Image analysis was conducted with ImageJ [23]. Tissue sections were 5 μm thick, comparable to the axial resolution of the SHG images, hence there was effectively a single layer of collagen in each sample, “auto-focused” with a maximum intensity projection of both the forward and backward image stacks. This produced a single image pair (forward scattered SHG “F”, and backwards scattered SHG “B”) for each imaged location. A maximum intensity projection of an 11 image scan taken with a closed microscope shutter was used to determine the background noise of the imaging system, which was then subtracted from each image. A common threshold (40 out of a maximum possible pixel count of 4095 a.u.) was initially determined by a blinded observer viewing ~30 image pairs and choosing the threshold that best distinguished pixels within fibers from those in the background. This single threshold was applied to each image to identify pixels within fibers by creating a pair of masks (one for F, one for B), in which all of the pixels above threshold were set to 1, and all of the pixels below threshold were set to zero. These masks were multiplied to create one “forward x backward mask” whose pixels were equal to 1 only when they were equal to 1 in both the forward and backward masks. The background subtracted F and B images were divided to produce an F/B image of the sample, which was multiplied by the “forward x backward mask”, and the average value of all nonzero pixels yielded the sample’s average F/B (Fig. 1).

Day-to-day variations in optical alignments were normalized by imaging a standard solution of FITC daily and applying a normalization factor for each detector pathway that rendered the signal from the standard FITC sample constant over time.

### FFT image analysis

FFT analysis was performed as previously described [17]. Specifically, the fast Fourier transform of each “F” image was generated via Matlab (MathWorks, Natick, MA). The FFT image was then binarized to include only the pixels with a value greater than 20. A linear regression was applied to the points using R Software (R Foundation, Vienna, AUS) and the R^2^ value was reported as a measure of the anisotropy of the overall orientation of the ensemble of collagen fibers in the image.

### Statistics

STATA, release 13 (StataCorp, Texas, USA) and Prism 5 software (GraphPad, La Jolla, CA) was used for statistical analysis. MFS was defined as the date of confirmation of a distant metastasis after symptoms reported by the patient, detection of clinical signs, or at regular follow-up. OS was defined as time until death, any cause, while patients who died without evidence of disease were censored at their last follow-up time.

PFS was defined as the time from start of tamoxifen treatment until a second line of treatment was needed, or until death. The relationship between the natural log of F/B (ln F/B) and survival rate was assessed using the Kaplan-Meier method and evaluated using the log-rank test for trend. Multivariate Cox proportional hazard analysis was applied to evaluate the prognostic value of the natural log of F/B, age, menopausal status, tumor size, tumor grade, ER, PgR and HER2 status. Differences were considered statistically significant when the 2-sided *p*-value was below 0.05.

## Results

### F/B and its relationship with patient and tumor characteristics

The median ln F/B of and interquartile range in all tumors was 2.228 (0.416) (Table 1). There was no significant association between ln F/B and age or menopausal status of the patient. There were also no significant correlations with tumor size, tumor grade, and HER2 status. In contrast, compared with steroid hormone-positive tumors, ln F/B was higher in ER- (*p* < 0.001) and PgR-negative tumors (*p =* 0.003), respectively (Table 1).

**Table 1:** Ln F/B and its association with breast cancer patient and tumor characteristics

Characteristics	No. patients (%)	Median levels (interquartile range)	*p*
All patients	221 (100 %)	2.228 (0.416)	
Age (years)			0.773^a^
≤ 40	33 (14.9 %)	2.160 (0.566)	
41–55	94 (42.5 %)	2.215 (0.410)	
56–70	70 (31.7 %)	2.291 (0.456)	
> 70	24 (10.9 %)	2.198 (0.327)	
Menopausal status			0.497^b^
Premenopausal	113 (51.1 %)	2.200 (0.447)	
Postmenopausal	108 (48.9 %)	2.250 (0.379)	
Tumor size			0.188^a^
pT1 (≤2 cm)	109 (49.3 %)	2.239 (0.356)	
pT2 (2–5 cm)	105 (47.5 %)	2.237 (0.505)	
pT3/pT4 (>5 cm)	7 (3.2 %)	1.830 (0.614)	
Tumor grade^c^			0.700^a^
I	37 (16.7 %)	2.207 (0.288)	
II	77 (34.8 %)	2.233 (0.366)	
III	101 (45.7 %)	2.264 (0.491)	
ER status			<0.001^b^
Positive	125 (56.6 %)	2.168 (0.407)	
Negative	96 (43.4 %)	2.311 (0.452)	
PgR status			0.003^b^
Positive	104 (47.1 %)	2.159 (0.392)	
Negative	117 (52.9 %)	2.302 (0.425)	
HER2 status			0.121^b^
Positive	26 (11.8 %)	2.299 (0.399)	
Negative	195 (88.2 %)	2.215 (0.432)	

^a^Kruskal-Wallis test

^b^Two-sample Wilcoxon rank-sum (Mann-Whitney) test

^c^Scarff-Bloom-Richardson grade (6 missing values)

### F/B and metastasis-free survival in breast cancer patients

**Fig. 2:**
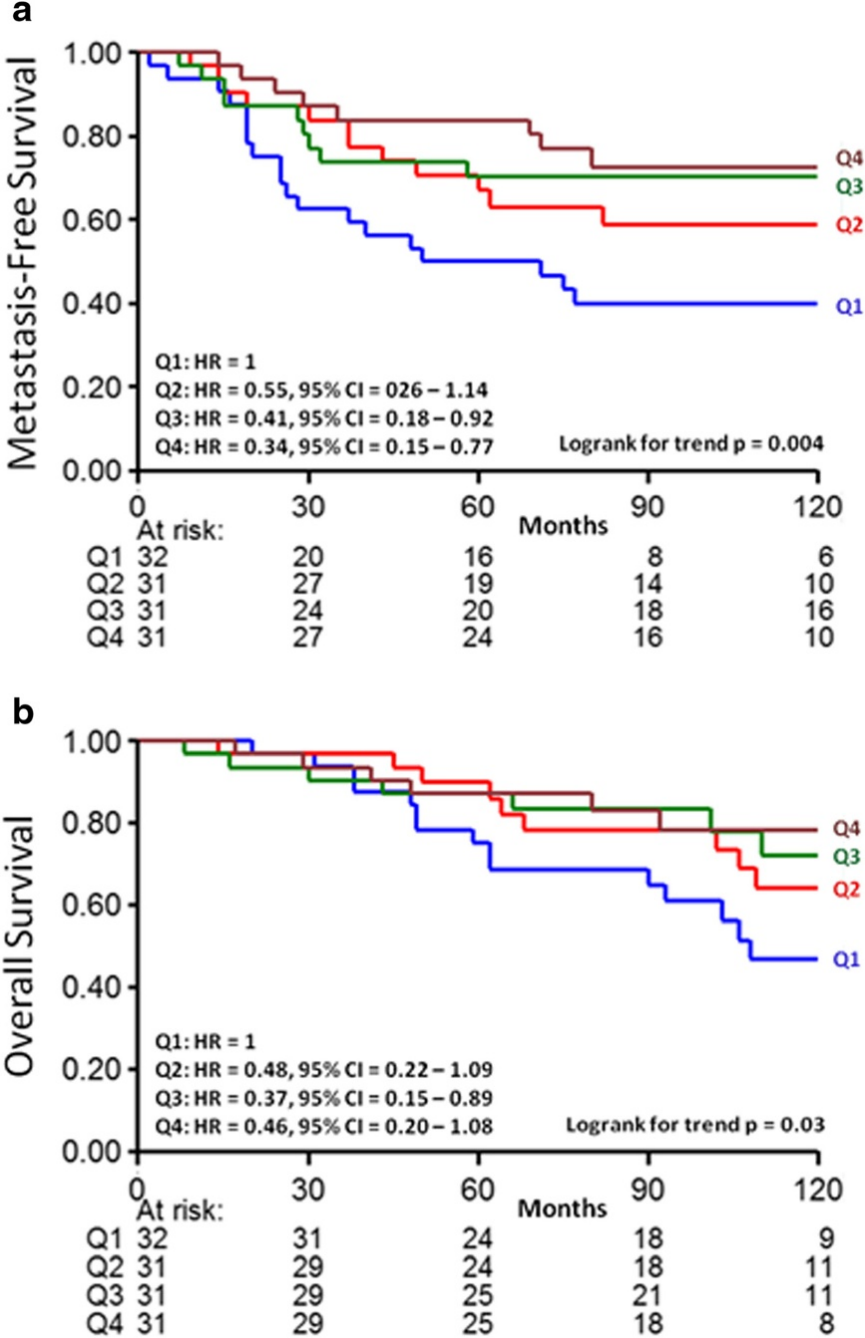
Metastasis-free (**a**) and overall survival (**b**) as a function of F/B in ER+, LNN breast cancer. The patients are divided in four equal quarters (Q1-Q4) based on their F/B tumor level. Patients at risk at various time points are indicated

Univariate analysis of the primary tumor ln F/B showed no statistically significant relationship between ln F/B and the length of MFS (Hazard Ratio, HR = 0.706; 95 % confidence interval, CI 0.351–1.422; *p* = 0.330) within the combined (ER+ and ER-) sample set. Because mechanisms of breast tumor progression varies based on ER status, and because ER+ and ER- tumors are biologically very different tumors [24, 25], we then analyzed the prognostic value of ln F/B in ER subgroups separately. Within the ER+ subgroup, in Cox regression analysis using ln F/B as a continuous variable there was a statistically significant relationship between the primary tumor ln F/B and MFS (HR = 0.23; 95 % CI 0.08–0.65; *p* = 0.005) (Table 2), but within the ER- population the relationship was not statistically significant (HR = 2.72; 95 % CI 0.8104–9.173; *p* = 0.105). The ER+, LNN patient samples were then divided into four equal quarters consisting of a high ln F/B (above 2.354: Q4), a low ln F/B (below 1.954: Q1), and 2 mid-range categories (range 1.954–2.168: Q2, and 2.168–2.354: Q3), and plotted in a Kaplan Meier curve (Fig. 2a). Patients with tumors with low F/B (Q1) showed the worst MFS, while those with high F/B (Q4) showed the best MFS. The 2-mid range categories (Q2 and Q3) showed an intermediate MFS (logrank trend *p* = 0.004). In Cox multivariate regression analysis for MFS in ER+ patients, corrected for the traditional prognostic factors age, menopausal status of the patient, tumor size, tumor grade, PgR and HER2 status, an increasing ln F/B was significantly associated with longer MFS (HR = 0.16; 95 % CI 0.05–0.55; *p* = 0.004) (Table 2).

**Table 2:** Cox univariate and multivariate regression analysis for MFS in 125 ER+ patients

	Univariate analysis			Multivariate analysis^a^		
Variable	HR	95 % CI	*p*	HR	95 % CI	*p*
Age						
41–55 vs 40 years	0.59	0.27–1.32	0.203	0.80	0.35–1.84	0.599
56–70 vs 40 years	0.56	0.25–1.26	0.159	0.41	0.13–1.34	0.140
> 70 vs 40 years	0.46	0.15–1.36	0.159	0.32	0.08–1.27	0.105
Menopausal status						
Post-vs premenopausal	0.98	0.55–1.73	0.938	2.46	0.89–6.84	0.083
Tumor size						
2–5 vs ≤2 cm	1.76	0.98–3.14	0.056	0.85	0.43–1.70	0.650
> 5 vs <2 cm	1.51	0.36–6.38	0.579	0.50	0.10–2.42	0.386
Tumor grade						
II vs I	3.15	1.30–7.61	0.011	2.76	1.10–6.92	0.030
III vs I	4.38	1.68–11.45	0.003	3.38	1.15–9.93	0.027
PgR status						
Positive vs negative	0.71	0.38–1.35	0.297	0.61	0.30–1.24	0.170
HER2 status						
Positive vs negative	4.06	1.71–9.65	0.002	3.67	1.10–6.92	0.009
Log of F/B ratio	0.23	0.08–0.65	0.005	0.16	0.05–0.55	0.004

^a^The multivariate model included 123 patients due to 2 missing values for tumor grade

### F/B and overall survival in breast cancer patients

Next we tested whether ln F/B of the primary tumor was also significantly related to OS in the ER+, LNN group of patients. Univariate Cox regression analysis showed that the primary tumor ln F/B was borderline statistically significantly related to OS (HR = 0.34; 95 % CI 0.11–1.03; *p* = 0.057). A logrank test for trend analysis of Kaplan Meier curves with ln F/B divided into Q1-Q4 shows a significant relationship between increasing ln F/B of the primary tumor and longer OS (Fig. 2b, *p* = 0.03). A multivariate Cox analysis of this data showed that ln F/B, when corrected for traditional prognostic factors, was borderline significantly related to OS (HR = 0.28; 95 % CI 0.07–1.10; *p* = 0.068) (Table 3).

**Table 3:** Cox univariate and multivariate regression analysis for OS in 125 ER+ patients

	Univariate analysis			Multivariate analysis^a^		
Variable	HR	95 % CI	*p*	HR	95 % CI	*p*
Age						
41–55 vs 40 years	0.49	0.21–1.17	0.108	0.61	0.25–1.52	0.289
56–70 vs 40 years	0.57	0.24–1.35	0.204	0.29	0.09–0.95	0.041
> 70 vs 40 years	0.33	0.09–1.26	0.105	0.20	0.04–0.95	0.043
Menopausal status						
Post- vs premenopausal	1.14	0.61–2.11	0.686	2.90	0.99–8.49	0.052
Tumor size						
2–5 vs ≤2 cm	1.25	0.66–2.37	0.494	0.56	0.26–1.20	0.137
> 5 vs ≤2 cm	1.66	0.39–7.11	0.492	0.75	0.15–3.72	0.720
Tumor grade						
II vs I	2.53	1.02–6.24	0.044	2.16	0.84–5.55	0.111
III vs I	5.02	1.89–13.36	0.001	4.88	1.64–14.56	0.004
PgR status						
Positive vs negative	0.51	0.26–1.01	0.055	0.48	0.23–1.01	0.054
HER2 status						
Positive vs negative	3.15	1.11–8.96	0.031	3.80	1.18–12.20	0.025
Log of F/B ratio	0.34	0.11–1.03	0.005	0.29	0.07–1.10	0.068

^a^The multivariate model included 123 patients due to 2 missing values for tumor grade

### Anisotropy and metastasis-free survival, as well as overall survival, in breast cancer patients

For comparison purposes we also evaluated whether the anisotropy of the orientation of the ensemble of collagen fibers in each image was predictive of metastasis free survival as well as overall survival. Univariate analysis of the primary tumor ln R value showed no statistically significant relationship between ln R and the length of MFS within the combined (ER+ and ER-) sample set (HR = 0.347; CI 0.077–1.557; *p* = 0.167), nor within the ER+ subpopulation (HR = 0.129; CI 0.015–1.074; *p* = 0.058), nor within the ER- subpopulation (HR = 0.945; CI 0.112–8.004; p=0.959). Likewise univariate analysis showed no significant relationship between ln R and length of OS within the combined sample set (HR = 0.567; CI 0.133–2.42; *p* = 0.443), nor within the ER+ subpopulation (HR = 0.213; CI 0.025–1.789; *p* = 0.154), nor within the ER- subpopulation (HR = 0.137; CI 0.203–9.18 l; *p* = 0.749).

### Tamoxifen treatment

**Fig. 3:**
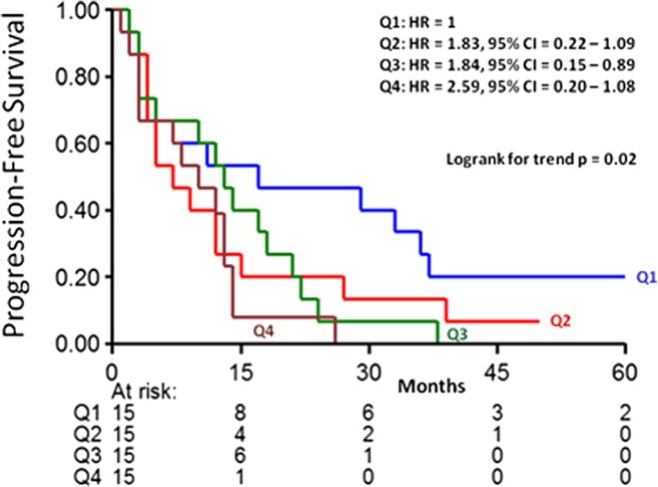
Progression-free survival as a function of F/B in ER+ recurrent breast cancer patients treated with tamoxifen. The patients are divided in four equal quarters (Q1-Q4) based on their F/B tumor level. Patients at risk at various time points are indicated

The previous studies were conducted in untreated patients in order to analyze the relationship between F/B of the primary tumor and tumor aggressiveness and pure prognosis. A subset of these patients did metastasize to a distant site and were then treated with tamoxifen as first-line monotherapy. Therefore we evaluated this subset of ER+ breast cancer patients to determine whether the F/B of the primary tumor was also significantly related to PFS after start of therapy for recurrent disease. The hazard ratio of the primary tumor ln F/B was 3.39 (95 % CI 1.22–9.37; *p* = 0.019) and the logrank test for trend analysis of Kaplan Meier curves in equal quarters showed a significant relationship (*p* = 0.02) between primary tumor ln F/B and PFS (Fig. 3). Interestingly, the trend in PFS (i.e. lower primary tumor F/B was associated with slower disease progression) was found to be the opposite of that observed in MFS and OS in the untreated ER+ patients (i.e. lower primary tumor F/B was associated with shorter MFS and OS times).

### Overall survival as a function of F/B in other solid tumor types

**Fig. 4:**
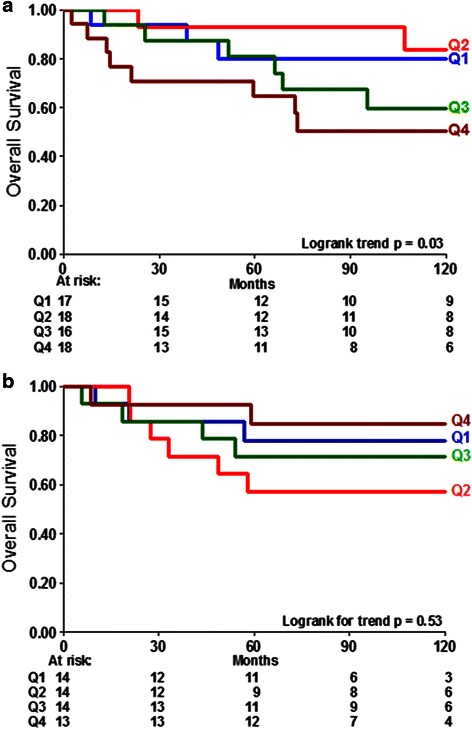
Overall survival of additional solid tumors as a function of F/B ratio. Overall survival in stage I colorectal adenocarcinoma (**a**) is significantly related to F/B of the primary tumor (*p* = 0.03). F/B of Stage I lung adenocarcinoma (**b**) is not significantly related to OS (*p* = 0.53). The blue line is Group 1 has the lowest F/B and the brown line is Group 4 has the highest F/B ratio. Patients at risk at various time points are indicated

Based on the significant relationships revealed in the breast cancer samples, we investigated colorectal and lung adenocarcinoma, other solid tumor types in which tumor cell/matrix interactions may significantly affect metastasis. Similar to ER+, LNN breast cancer patients, stage I colorectal and lung adenocarcinoma are subsets of patients where there is a clinical need to assist the physician in deciding the appropriate level of treatment for the patient. In stage I colorectal adenocarcinoma there was a significant relationship between the F/B of the primary tumor and patient OS (Fig. 4a). Notably, the observed trend (i.e. a lower F/B was associated with longer OS) was the opposite of the trend observed in the untreated ER+, LNN breast cancer samples, suggesting a different mechanistic relationship between metastasis and collagen fiber microstructure. In contrast, stage I lung adenocarcinoma showed no significant relationship between the F/B of the primary tumor and OS (Fig. 4b). This suggests that not all solid tumors undergoing metastasis elicit identical collagen restructuring or utilize identical mechanisms relating metastatic ability and collagen microstructure.

## Discussion

Currently the ER+, LNN breast cancer population suffers from over-treatment as many patients receive chemotherapy even though metastatic disease never would have arisen. As such, there is a pressing need to improve clinicians’ ability to predict which tumors are likely to metastasize in this population. Current methods to predict metastasis are “cell focused”, using quantification of gene and protein expression levels, or cellular morphology and cell-cell interactions [7–9, 11]. However, the process of metastasis is a complex interplay between tumor cells and their microenvironment, including the extracellular matrix [26, 27]. Therefore we explored the prognostic ability of a “matrix focused” measurement, the SHG F/B of the primary tumor.

Studies demonstrating that SHG imaging can differentiate healthy and tumor tissue in ovarian [28], basal cell [29], and pulmonary cancers [30], have established that SHG is an intrinsic signal which reports on clinically relevant properties of the tumor extracellular matrix. We recently applied this methodology in breast cancer, demonstrating that the simple intensity-based SHG F/B is significantly different amongst different breast tumor types [18] hence we explored its ability to predict metastatic outcome. For comparison we also explored the ability of simple FFT analysis of fiber anisotropy. While the two method report upon different structural properties (F/B is affected by fibril diameter, spacing, and disorder within a fiber [14–16], while anisotropy reports on the overall orientation of ensembles of fibers in an image [17]) both are easily automatable analyses. In the current work, we demonstrate that F/B analysis of the primary tumor is a prognostic indicator in the ER+, LNN population. Unlike the ER- or ER+ node-positive patients, in whom adjuvant chemotherapy is universally applied, the choice of whether or not to prescribe adjuvant chemotherapy (e.g. doxorubicin, fluorouracil, etc.) in addition to tamoxifen for ER+, LNN patients is not easily apparent. Hence this is a population with a significant over-treatment problem requiring improved prognostic indicators. Our results suggest that SHG F/B from the primary tumor specimen may offer insight into eventual metastatic outcome of the patient and thus may help reduce over-treatment. Currently, predicting the time to metastasis in this population is primarily facilitated by histopathology and by genetic screens. These genetic screens quantify gene expression in cells within the tumor, including both the tumor and stromal cells. The SHG-based method demonstrated here may be highly complementary to those genetic screens, as it derives its information from the structure of the extracellular matrix in the primary tumor, rather than from the tumor cells themselves. SHG imaging has been used previously to predict breast cancer survival times, however these studies focused on analysis of morphological information from collagen images, requiring trained pathologists to score the orientation of collagen fibers in images [31]. Furthermore, the majority of that sample population was lymph node positive, while our study focuses on the LNN population, in which the key decision on adjuvant chemotherapy must be made and for whom the risk of over-treatment is high.

Based on the important role that tamoxifen plays as a treatment in almost all ER+ breast cancer patients, after identifying the significant relationship between F/B and patient outcome in untreated patients, we were interested in exploring the prognostic capability of F/B to determine the effects of tamoxifen on patients with recurrent tumors. Our results revealed that F/B as measured on the primary tumor was prognostic of PFS after patients who developed a metastasis at a distant site were treated with tamoxifen. Interestingly, the actual relationship between F/B and outcome displayed a trend that was opposite to that in the MFS and OS findings from untreated ER+ patients: In tamoxifen treated recurrent ER+ patients a high F/B was associated with a faster rate of progression, whereas in untreated ER+ patients a high F/B was associated with improved MFS and OS. Tamoxifen is an ER antagonist, indicating this contrast between tamoxifen treated ER+ tumors and untreated ER+ tumors could be due to the roles of ER in tumor progression. To explain this pattern of relationships between recurrence and F/B in ER+ tamoxifen treated tumors, as opposed to untreated ER+ tumors, we therefore hypothesize that differences in primary tumor collagen microstructure may indicate differences in the mechanism by which tumor cells spread, which has the effect of altering susceptibility to later treatment. In an ER+ primary tumor with a low F/B, cells spread into vasculature and to secondary locations, and upon tamoxifen administration these secondary tumors are effectively treated. In an ER+ primary tumor with a high F/B ratio, tumor cells metastasize via different mechanisms which decrease the tumor cell sensitivity to tamoxifen treatment.

The results demonstrating another significant relationship between F/B of the primary tumor and OS, in stage I colorectal adenocarcinoma, indicate that the mechanisms relating metastasis to collagen microstructure may be similar between breast cancer and other solid tumors. Analyzing collagen structure in colorectal adenocarcinomas may thus aid in predicting the OS rates in patients, consequently helping to tailor the choice of chemotherapy in that tumor type as well, with low-risk patients receiving no treatment and high-risk patients being considered for neoadjuvant chemotherapy (fluorouracil, etc.). The fact that the primary tumor F/B was not predictive of metastasis in stage I lung adenocarcinoma provides support for the idea that multiple mechanisms of tumor metastasis may exist, involving differential interplay between tumor cells and matrix microstructure. These alternative mechanisms could be the result of different levels of fibrous tissue in the tissues of origin, (e.g. collagen density is high in breast and colon but not in lung tissue). In the future it may therefore be beneficial to investigate the relationship between primary tumor F/B and metastatic outcome in other solid tumors that are typically characterized as more fibrous, such as pancreatic cancer.

## Conclusions

In summary, we have identified the F/B, a simple and easily automated, intensity-based measurement as an independent prognostic indicator of metastatic outcome in ER+ LNN breast cancer patients. Furthermore, escaped tumor cells with a low F/B at the primary site show a better responsiveness to tamoxifen treatment of the recurrence, indicating a possible mechanism by which collagen structure at the primary site affects sensitivity to treatment. The primary tumor F/B is also prognostic in stage I colon adenocarcinoma, suggesting this assay may be useful in multiple types of solid tumors. By imaging the tumor “soil” this method provides information complementary to that offered by current cell-focused techniques, and therefore in combination with those methods may improve prediction of recurrence and hence reduce over-treatment.

## Abbreviations

ER+: Estrogen receptor positive
ER-: Estrogen receptor negative
F/B: Ratio of the forward-to-backward emitted SHG signals
FITC: Fluorescein isothiocyanate
H&E: Hematoxylin and eosin
HR: Hazard ratio
LNN: Lymph node-negative
MFS: Metastasis-free survival
OS: Overall survival
PFS: Progression-free survival
PgR: Progesterone receptor
SHG: Second harmonic generation
TMA: Tissue-microarray
